# Women in Lymphoma: A 4‐year journey in promoting gender equity

**DOI:** 10.1002/hon.3183

**Published:** 2023-05-25

**Authors:** Judith Trotman, Ann LaCasce, Wendy Osborne, Anna Steiner, Eliza Hawkes, Carla Casulo, the Women in Lymphoma Steering Committee, Florence Broussais, Carla Casulo, Kate Cwynarksi, Paola Ghione, Eliza Hawkes, Justine Kahn, Sharyn Kurtz, Ann LaCasce, Kim Linton, Carolina Mahuad, Monique Minnema, Loretta Nastoupil, Wendy Osborne, Astrid Pavlovsky, Michelle Poon, Clémentine Sarkozy, Laurie Sehn, Soni Smith, Anna Sureda, Carrie Thompson, Judith Trotman, Julie Vose

**Affiliations:** ^1^ Concord Repatriation General Hospital University of Sydney Concord New South Wales Australia; ^2^ Dana‐Farber Cancer Institute Boston Massachusetts USA; ^3^ Department of Haematology Freeman Hospital Newcastle Hospitals University of Newcastle upon Tyne Newcastle upon Tyne UK; ^4^ Women in Lymphoma Lymphoma Australia Brisbane Queensland Australia; ^5^ Olivia Newton‐John Cancer Research & Wellness Centre Austin Health Melbourne Australia; ^6^ Monash University Melbourne Victoria Australia; ^7^ University of Rochester Medical Centre Rochester New York USA

**Keywords:** gender equity, lymphoma, women

## 1 INTRODUCTION: THE NEED FOR CHANGE

Gender diversity, equity and inclusion (DEI) in academic and clinical medicine is acknowledged as more than just the ‘*right thing to do*’. It has led to improvements in innovation, leadership quality, as well as workplace culture and retention for all genders.^1^ In hematology and oncology, recent progress includes the first female presidents of large international organizations such as the American Society of Hematology (ASH), American Society of Clinical Oncology (ASCO), British Society of Hematology (BSH) and European Society of Blood and Marrow Transplantation (EBMT). Yet in our own field of lymphoma, despite gender parity in medical training for decades, women are underrepresented in leadership roles due to continued failures of system structures, and inherent often unconscious biases.^2^, ^3^ Lymphoma ‘*manels*’ at some international conferences persist, and there is a visible minority of female speakers and first or senior investigators/authors in academic presentations and publications. Few women have a ‘*seat at the table*’ in editorial and advisory boards, and limited leadership of industry clinical trials continues, despite a large pipeline and talent pool of female lymphoma experts globally.

As counterpoint to this Women in Lymphoma (WiL) is delighted to be invited by the Editor of Hematological Oncology to present this Special Issue, showcasing a suite of expert updates in lymphoma, the first ever to be authored exclusively by women. We also share this introductory article describing the formative years of WiL, inspired and informed by the authors' lived experience with near parity in gender representation in clinical practice, while remaining in a significant minority group within lymphoma leadership. We trust this article and two accompanying manuscripts, one written by three senior European WiL reflecting on their career challenges and opportunities, and a perspective from the inaugural male Chair of WiL's Change Champions Committee, educate and empower both women and men in lymphoma to address this inequity, to hasten academic progress and advances for our patients.

## 2 THE FORMATION OF WiL

Women in Lymphoma, (WiL), www.womeninlymphoma.org is a global organization founded in 2019 by a group of female international lymphoma leaders. The catalyst for WiL was an advertised “*manel*” at an ICML 2019 corporate symposium. At a meeting where >50% of the several thousand attendees were female, from both a social justice and a business lens, such *manels* were wrong, promoting unconscious bias and sidelining women in lymphoma care and research.^4^, ^5^, ^6^ The following invitation was emailed to women in lymphoma through informal networks:

- *Are you passionate about your lymphoma research—in basic, translational, clinical trials or implementation science?*
- *Have you sought to build your academic career but lacked the mentors to support you?*
- *Are you searching for inspiration and tactical advice to contribute more fully within your research group?*
- *Are you seeking to enhance your lymphoma group's organisational culture by promoting productive diversity?*
- *Do you understand the data on implicit bias in medical research communities?*

*You are welcomed to join a collegial group of your female peers gathering at ICML. We will be sharing ideas to build a network of support and advocacy for greater leadership of women in lymphoma research*.

Thus, over 70 women attended a planning meeting at ICML 2019 and formed the initial membership for Women in Lymphoma (WiL), a global collaborative alliance of lymphoma clinicians, researchers & educators committed to support and advocate for greater leadership of women. WiL defined their Vision for “*greater leadership of women in lymphoma globally”,* along with the tagline of “*Where there's a WiL, there's a way!”*

Throughout Zoom meetings in 2019 WiL established a Charter and defined a threefold mission:

1. To **inspire and empower WiL** to greater (and more visible) leadership in lymphoma care, research and teaching.
2. To **map the metrics** of such engagement.
3. To be a **collective voice to advocate** for equal representation in lymphoma leadership on behalf of women in lymphoma and all lymphoma clinicians, researchers and patients.

Women in Lymphoma is led by an executive group of the current Chair, immediate‐past Chair, Chair‐Elect and Secretary, supported by a Steering Committee, (SC) responsible for initiatives aligned with WiL's mission. This SC ‐ formed initially in 2019 by 16 female lymphoma specialists from Australia, Argentina, Canada, France, The Netherlands, Singapore, UK & USA ‐ was increased in 2020 to 23 members after a specific recruitment campaign for early‐career/emerging lymphoma leaders and for broader global membership. In expanding our SC membership to include “junior” or emerging WiL leaders we provide a supportive space to articulate the needs of early career WiL who face different systemic challenges in the modern era. The issue of global representation and intersectionality is also important for WiL, needing the engagement of members from a diverse ethnic and social background. However, in a world where addressing gender equity creates varying levels of discomfort across different cultures, we resolved to incrementally increase the representation of women of color and minority groups globally, without expecting these women to shoulder too great a burden of advocacy for change. To achieve global impact we recognized that, as an embryonic organization, we needed to first effect sustained change within countries with well‐established and accepted gender equity programs.

WiL grew rapidly with a membership of 200 within the first year and then to 950 by May 2023. The administrative activities inherent to a rapidly growing start‐up organization grew, and while it was important for the SC to provide intellectual input and direction to WiL's mission we didn't want these leading WiL, with heavy clinical, academic, and personal caring responsibilities, exacerbated by the COVID pandemic,^7^ to be burdened by the administrative work of documenting and executing these ideas. To that end, with metrics demonstrating the early impact of WiL, Executive Administrator support was sourced from two Australian lymphoma charities. This business and governance support is contingent on WiL's independent fundraizing for educational initiatives via unrestricted grants from pharmaceutical companies as acknowledged below.

## 3 MISSION 1: INSPIRING AND EMPOWERING WiL

*“You can be what you can see”.*

Throughout 2019–2020 WiL received emails of concern about the low representation of female speakers at educational fora, despite their heavy educational load in their own hospitals and universities.^8^, ^9^ WiL responded in two ways: creating a freely available virtual educational platform for all, unique in its restriction to female speakers; and sending constructive correspondence to conference organizers about gender equity.

As the COVID‐pandemic shut down conferences in 2020, the value in providing a supportive virtual global platform to amplify the voice of women as educators was even more apparent. WiLing Wednesday's Webinars: *“by women, not just for women”*, were conducted via Zoom, at 4 PM in the time zone of the presenters across six continents https://womeninlymphoma.org/events‐%26‐news. By April 2023, thirty‐seven lectures delivered by 116 female experts across eight webinar series have been watched live by approximately 50% of the registered 3063 (286–515 per series) attendees, and in delayed viewing by 1022 (125–166 per series) per month. Having covered every lymphoma histology in 2020–2023, in 2024 updated webinars will commence again with Diffuse Large B‐cell Lymphoma. In more recent series, WiL introduced critical skills teaching, commencing with career development lectures from senior US WiL. A subsequent webinar with career reflections from legendary European WiL, who wrote an accompanying manuscript in this Special Issue,^10^ provided complementary insights from different cultural settings, of value for junior lymphoma clinicians and researchers of any gender. The first attempt to run this webinar was abandoned after Zoom bombing with a high‐volume tirade of obscene language. This is WiL's sole experience of gendered backlash in 4 years of operation, and now registration for all WiL Webinars is required. To engage with and inspire its growing membership, WiL publishes quarterly newsletters to highlight WiL initiatives, congratulate WiL members on academic promotions and achievements, and profile published data in gender equity.

## 4 MISSION 2: MAPPING THE METRICS

“*Holding educational and research organizations to account*”.

### 4.1 Educational fora

A common argument made for the lack of equitable female representation in global educational fora is that few women reach high levels in academia, with a smaller pool of experts available to speak. The slower rate of academic advancement for women is well recognized^11^ and current US estimates of women holding 14% of Professorial titles in medicine are sobering, when the last time there was as low a proportion of females in medical school was 1972.^12^ In keeping with the principles of equity and recognizing that change requires acknowledgment of and adjustment to imbalance, WiL believes that lymphoma conference organizers have both a moral responsibility and business incentive to search out female educators to facilitate a better gender balance. Presenting at such international fora is one of the currencies by which academic promotion is secured. Furthermore, as acknowledged earlier, women carry a significant load of education portfolios in hospitals and universities despite their marked under‐representation in direction of research portfolios.

Recognizing the importance of not just mapping but also seeking to change inadequate metrics, WiL has written formally to the organizing committees of conferences documenting the male:female imbalance in educational symposia disproportionate to the demographics of clinical practice and conference attendees. Such constructive correspondence with haematology organisations was designed to both highlight opportunities for change and acknowledge successful measures in moving toward equity. In future these metrics will be publicly charted on a revamped WiL website: https://womeninlymphoma.org.

One notable imbalance ‐ brought to SC attention by junior WiL in 2020 ‐ was the European School of Haematology (ESH) “*How to diagnose and treat lymphoma”* course. Having sent WiL correspondence documenting that only 1/42 (2.4%) of speakers in the draft program of the 2020 course was female, it was gratifying to witness ESH as being highly responsive to change, with 17/42 (40.5%) female speakers at the 2022 course.

In 2020 correspondence was also sent to the Local Organizing Committee (LOC) of the International Congress on Malignant Lymphoma (ICML), the key international lymphoma scientific meeting held biannually, noting their 50% female attendance and urging attention to fair gender representation amongst invited speakers and chairpersons in future meetings. At the 2021 meeting females were 6/21 (28.6%) invited speakers, and 1/11 (9.1%) ‘*Meet the Professor’* presenters, and 29/88 (33.0%) of the oral abstract presenters. Since then, the LOC ‐ previously 100% male ‐ has 3/14, (21%) female members, and 5/25 (20%) of the ICML Advisory Board are female. At the 2023 ICML meeting, 4/12 (33%) ‘*Meet the Professor’* presenters are women, as are 24/72 (33.3%) Session Chairs, 23/86 (26.7%) invited speakers, and 39/111 (35.1%) of oral abstract presenters. Reflecting a genuine commitment to promoting greater diversity, equity and inclusiveness (DEI) the three Perspective articles in this invited special issue of Haematological Oncology are timed for online release in conjunction with the 2023 ICML meeting. The seven histology specific articles authored exclusively by WiL are currently undergoing peer review.

Two standout organisations to whom WiL wrote letters acknowledging their highly visible, broad inclusion of women in education, were the BSH, where the Lymphoma Special Interest Group has had equal gender representation at all educational meetings since its formation in 2017, and ASH. At the ASH 2020 Congress there were an unprecedented 10/17 (58.8%) female Chairs and speakers in the Lymphoma Educational Symposia, with notable inclusion of women of minority ethnicities. Additionally, an analysis of the 2020 ASH oral abstract speakers provided WiL with baseline metrics for further initiatives to increase female leadership in lymphoma research. While 11/32 (34.3%) lymphoma oral session moderators were women, there were only 15/82 (18.3%) female first authors, and 15/82 (18.3%) female last authors. In 2021 a higher proportion of oral abstracts, 35/111 (31.5%) had a female first author but there were still only 16/111 (14.4%) female senior authors. In 2022 women were 5/11 (45.5%) educational symposia presenters and 19/45 (42.2%) lymphoma oral session moderators, with a gratifying higher proportion of women both as oral first authors 60/156 (38.5%) and last authors 48/156 (30.8%). Of interest in 2022, male last authors (*n* = 108) collaborated equally with male and female first authors in proportion to their representation 67/96 (70%) and 41/60 (68%). This data suggests an encouraging change in the traditionally homophilic patterns of male research collaborations.^13^ This is particularly important as the pipeline of junior female lymphoma researchers continues to grow and mentorship of these women can't be left solely to the smaller cohort of senior women in our field. Nonetheless, the rise in the proportion of senior (last author) women in oral lymphoma abstracts from 18% in 2020 to 31% in 2022 is impressive and warrants ongoing monitoring for a sustained increase. In their response to WiL's correspondence, both BSH and ASH noted their appreciation of the monitoring of an external organization, observing the actions of conference organisers in their DEI initiatives.

The ASCO meeting has a much smaller lymphoma program but in 2021 women were again well represented in educational sessions: 2/3 66% of Chairs and 5/9 (55.5%) speakers. For the oral abstracts women were 1/9 (11.1%) first and 3/9 (33.3%) last authors. In 2022 there was a 50:50 spilt in educational presenters and women represented 3/9 (33.3%) of oral abstract presenters. Together, the ICML, ASH and ASCO abstract data suggests that positive DEI initiatives within formal haematology/oncology societies are not replicated at the trial sponsor level, whether university/hospital, cooperative group or industry led.

Finally, a short report written by WiL outlining the less than one third female representation as authors of lymphoma‐specific reviews, commentaries and editorials in eight leading journals since 2017 is under journal review.

### 4.2 Cooperative group and industry research

Mapping the above metrics in abstract presentation prompted two further initiatives; the design and distribution of a survey to cooperative clinical trial groups globally, and reaching out to women in industry to better understand the notably homophilic patterns of clinical research leadership in the lymphoma pharmaceutical industry.

After the successful implementation of systematic measures to address gender inequity in leadership of the Australasian Leukemia Lymphoma Group (ALLG) in 2020, the WiL SC has commissioned the ALLG CEO to conduct a global survey of cooperative trial groups working in lymphoproliferative diseases to map the current status of gender diversity in their groups. We encourage every cooperative group to participate confidentially to this international survey https://www.surveymonkey.com/r/TBPKVY8.^14^ To date nine groups have responded and aggregate data will be presented once we have responses from at least half the estimated *n* = 25 cooperative groups conducting lymphoma trials globally.

There are ongoing international efforts to increase female representation at C‐suite executive and Board level in the business community. Similarly, WiL's nascent industry sub‐committee established in 2023 will consider initiatives to improve the representation of women in senior biopharmaceutical industry roles, as well as in addressing how industry engages in a transparent and equitable basis with academic women in lymphoma, both in funded research and advisory boards.

## 5 MISSION 3: COLLECTIVE ADVOCACY

An important aspect of WiL as a platform for collective advocacy is to minimize criticisms for individual women who defy gender stereotypes. It is important to address the recognized perceptions that female leaders are less legitimate and minimize backlash against gender‐norm violations that will occur with choosing, for example, a talented female chief investigator, or research group leader ‘*in lieu*’ of a talented male.^15^ Gender equity is not a female issue but a societal one, and as WiL became firmly established in 2020 the SC recognized that WiL needed to facilitate the powerful advocacy of men, stepping up beside women to accelerate gender equity. Based on an analysis of the Male Champions of Change corporate initiative: https://championsofchangecoalition.org ^16^ the WiL Change Champions Committee was established, comprised of five WiL SC members and seven invited male lymphoma leaders with a track record of mentoring women. The initiatives of the CCC are summarized in an accompanying article^17^ including drafting a panel pledge encouraging individuals to make a personal commitment to gender balance and inclusion in panels and presentations, and producing sample scripts: https://womeninlymphoma.org/about‐us ^18^ for men and women to use in advocating for gender diversity, and in particular avoiding the creation of ‘*manels*’.

## 6 THE FUTURE FOR WiL

WiL's rapid membership growth over 4 years and the many, often intangible, achievements to date reflect the commitment of almost one thousand women and men to acknowledge their own personal biases and address gender inequity. The multiple‐mentoring model created within the WiL SC has established a powerful collaborative network advocating for and effecting change within our discipline. A future challenge for WiL will be to extend the value of this mentoring model to the broader membership. Our WiLing Wednesdays Webinars will continue to educate and provide career development support for both genders, and the primarily young WiL who attend are hopefully inspired to *‘be what they can see*’. We seek to enhance the capacity of both women and men, the #*heforshe,* who genuinely wish to conduct their teaching, research and lymphoma practice in an inclusive way, recognizing gendered and individual differences in learning and teaching and leadership styles. As evidence‐based clinicians, WiL plan to engage more systematically with experts in the gender equity sphere, including those in ASH and other hematology organizations, to build on what is now a robust platform for change. Cultural appetite for change: how much, how soon, and how widespread, will vary globally but we have demonstrated “*Where there's a WiL there is a way*”. WiL urges the global lymphoma community to join us in addressing our unconscious bias and dismantling structural sexism in lymphoma for the betterment of all lymphoma clinicians, researchers and patients. Collectively we continue to strive for an era of such sustained inclusivity and productive diversity in lymphoma leadership that WiL need exist no more.

## FUNDING INFORMATION

WiL has received funding support from the following pharmaceutical companies in unrestricted educational grants for the WiLing Wednesdays webinars: AstraZeneca, Beigene, BMS‐Celgene, Janssen, MSD, Mundipharma, Roche and SeaGen.

## CONFLICT OF INTEREST STATEMENT

The authors have no relevant conflicts of interest.

### PEER REVIEW

The peer review history for this article is available at https://www.webofscience.com/api/gateway/wos/peer-review/10.1002/hon.3183.

## Data Availability

The data that support the findings of this study are openly available in ASH, ICML and ASCO conference websites.
